# High Classification Accuracy of a Motor Imagery Based Brain-Computer Interface for Stroke Rehabilitation Training

**DOI:** 10.3389/frobt.2018.00130

**Published:** 2018-11-29

**Authors:** Danut C. Irimia, Rupert Ortner, Marian S. Poboroniuc, Bogdan E. Ignat, Christoph Guger

**Affiliations:** ^1^g.tec Medical Engineering GmbH, Schiedlberg, Austria; ^2^Gheorghe Asachi Technical University of Iasi, Iasi, Romania; ^3^Grigore T. Popa University of Medicine and Pharmacy, Iasi, Romania; ^4^Guger Technologies OG, Graz, Austria

**Keywords:** brain-computer interface, motor imagery, stroke, rehabilitation, classification accuracy

## Abstract

Motor imagery (MI) based brain-computer interfaces (BCI) extract commands in real-time and can be used to control a cursor, a robot or functional electrical stimulation (FES) devices. The control of FES devices is especially interesting for stroke rehabilitation, when a patient can use motor imagery to stimulate specific muscles in real-time. However, damage to motor areas resulting from stroke or other causes might impair control of a motor imagery BCI for rehabilitation. The current work presents a comparative evaluation of the MI-based BCI control accuracy between stroke patients and healthy subjects. Five patients who had a stroke that affected the motor system participated in the current study, and were trained across 10–24 sessions lasting about 1 h each with the recoveriX system. The participants' EEG data were classified while they imagined left or right hand movements, and real-time feedback was provided on a monitor. If the correct imagination was detected, the FES was also activated to move the left or right hand. The grand average mean accuracy was 87.4% for all patients and sessions. All patients were able to achieve at least one session with a maximum accuracy above 96%. Both the mean accuracy and the maximum accuracy were surprisingly high and above results seen with healthy controls in prior studies. Importantly, the study showed that stroke patients can control a MI BCI system with high accuracy relative to healthy persons. This may occur because these patients are highly motivated to participate in a study to improve their motor functions. Participants often reported early in the training of motor improvements and this caused additional motivation. However, it also reflects the efficacy of combining motor imagination, seeing continuous bar feedback, and real hand movement that also activates the tactile and proprioceptive systems. Results also suggested that motor function could improve even if classification accuracy did not, and suggest other new questions to explore in future work. Future studies will also be done with a first-person view 3D avatar to provide improved feedback and thereby increase each patients' sense of engagement.

## Introduction

A brain-computer interface (BCI) enables a direct communication pathway between the brain and external devices. The users perform certain mental tasks that entail distinct brain patterns of brain activity, and automated tools can detect that activity and thereby provide communication. Most BCIs are noninvasive systems that rely on the electroencephalogram (EEG). Noninvasive BCIs typically rely on one of three mental tasks, entailing motor imagery (MI) or selective attention to transient or oscillating stimuli (Wolpaw and Elizabeth, 2012).

Most BCIs sought to provide communication for severely disabled users. However, recent analyses and commentaries have addressed promising new goals and user groups, including motor rehabilitation for stroke patients (Prasad et al., 2010; Wolpaw and Elizabeth, 2012; Allison et al., 2013; Brunner et al., 2015). In this approach, the users perform mental imagery tasks that are well-established in motor rehabilitation therapy, such as imagination of left or right hand dorsiflexion. In typical MI training, users are instructed to imagine the movement, without any means to confirm that the user is indeed performing the desired mental activity. The BCI technology can provide an objective tool for measuring MI, thus providing the possibility of “closed-loop” feedback. Since closed-loop feedback that is effectively paired with the desired mental activity is a critical facet of any feedback system (Neuper and Allison, 2014), the “paired stimulation” (PS) made possible through motor-imagery BCI research could improve therapy outcomes. Recent research from several groups has supported this hypothesis (Ang et al., 2011; Pichiorri et al., 2011, 2015; Ortner et al., 2012; Luu et al., 2015; Sburlea et al., 2015; Soekadar et al., 2015; Remsik et al., 2016; Sabathiel et al., 2016; Serrano et al., 2017). Furthermore it is known that a closed feedback loop increases the users performance (Wolpaw et al., 2002).

Within the PS approach, the classifiers must accurately interpret a user's MI. Thus, even though the overall goal of PS is not communication but rehabilitation, classification accuracy is relevant. Ineffective classification could mean that the system provides rewarding feedback when users are not imagining the correct movement, or the other way round: provide no feedback even if the patient is doing correct imagination. Both cases reduce the advantage of PS over conventional therapy, where, for example, the patient has to imagine a limb movement while a therapist or assistive device is mobilizing that limb. Furthermore, a reduced accuracy would correspond to a lower training intensity and of course the treatment time of stroke patients should be optimally used. Finally, the BCI accuracy can serve as performance feedback for patients to motivate them to participate in the training and to try to reach optimal performance. The inclusion of FES to stroke rehabilitation could further improve classification accuracy (Do et al., 2011).

In MI BCIs for stroke rehabilitation or communication, accurate classification relies on the automated signal processing tools used. Guger and colleagues tested the accuracy of a MI-based BCI in 2003 on 99 healthy users. Ninety-Three Percent of these users reached an accuracy level better than 60% (Guger et al., 2003). Due to constraints in available time for each subject and the usage of passive EEG electrodes, that study used an easy setup with two bipolar channels. More advanced approaches take advantage of a high number of EEG channels to increase accuracy. The method of common spatial patterns (CSP) is often used to create subject specific spatial filters for these setups. Numerous articles explored classification accuracy with CSP with different subjects (Vidaurre and Blankertz, 2010; Ortner et al., 2015). A study with 64 EEG channels from 20 healthy young adults (mean age 23.5) while they imagined left or right hand movement within one 60-min BCI session achieved a mean accuracy rate of 72.4%, while the mean maximum accuracy rate was 80.7%.

The current pilot study explores stroke patients' BCI classification accuracy by using a MI BCI with continuous cursor feedback and additional functional electrical stimulation (FES). The afferent feedback provided by FES temporally coupled with task-related motor execution could facilitate brain plasticity (Quandt and Hummel, 2014). A second goal is to see if the MI performance of stroke patients gets better with training, and we also investigate improvements in motor function resulting from training. In a meta-analysis the accuracy is compared to data from healthy controls assessed in one of our earlier study (Ortner et al., 2015) where we used the same paradigm and experimental setup, except the FES.

## Materials and methods

The study was approved by the institutional review board of the Rehabilitation Hospital of Iasi, and all patients signed an informed consent and an authorization for release of photographs and videos before the start of the study.

**Figure 1:**
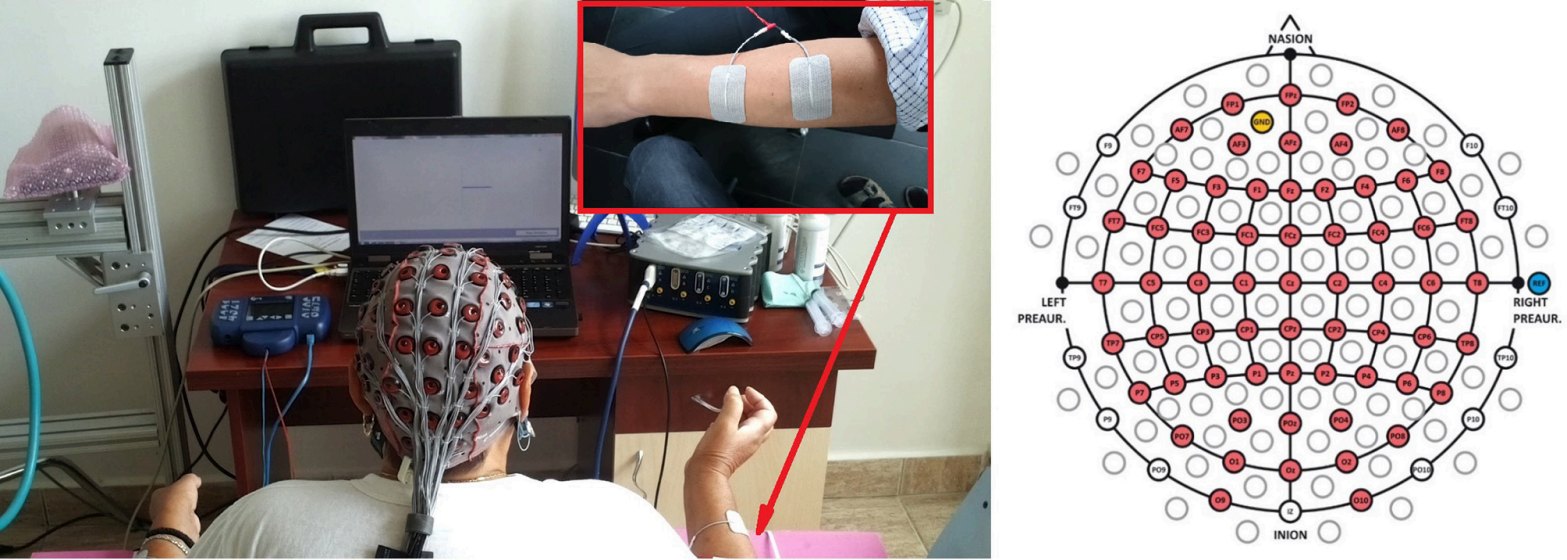
The **left** panel presents the experimental setup with a patient sitting in front of a computer screen wearing a 64 channel EEG cap. The EEG signals are decoded by a g.HIamp biosignal amplifier, and FES is provided through an 8-channel neurostimulator. The FES electrodes are assembled on both forearms to perform wrist extension. The written and informed consent has been obtained from the depicted individuals for the publication of their identifiable image. The **right** panel displays the electrode placement used in this study, according to the 10–20 international system.

All patients used an early version of the recoveriX system, consisting of a computer, a FES device, a biosignal amplifier with active EEG electrodes, and a feedback screen for the patient. Each patient was seated one meter in front of a computer monitor. The electrodes for functional electrical stimulation (FES) were placed over the posterior muscles of the forearms to induce wrist and finger extension upon stimulation. The complete setup is shown in Figure 1.

During hospitalization, in addition to the recoveriX training, all patients performed 30 min of conventional rehabilitation therapy per working day. This therapy consisted of passive and active movements performed individually or in larger groups, under the supervision of a therapist.

### Patient description and inclusion and exclusion criteria

Five patients participated in the study (mean age: 60 years, 3 males, 2 females). Table 1 summarizes information about the patients in our study. They all suffered an ischemic stroke in the territory of the sylvian artery (three of them had cortical strokes and two deep lacunary strokes).

**Table 1:** The coded list of participants in the study, the time interval between the stroke occurrence and the start of recoveriX training, description of movement difficulties and number of recoveriX sessions.

**Patient**	**Time between the stroke and recoveriX training/stroke location**	**Difficulties right hand**	**Difficulties left hand**	**Sessions**	**Age**	**Sex**
P1	12 months/Cortical-lacunary in the deep territory of the MCA	No	Yes (limited opening and grasping)	14	64	M
P2	1 month/Cortical -lacunary in the deep territory of the MCA	Yes (limited movement)	No	24	61	F
P3	4 years/Cortical–superficial sylvian territory	No	Yes (paralyzed hand)	10	40	F
P4	4 months/Cortical-superficial sylvian territory	Yes (limited hand movement)	No	22	69	M
P5	3 months/Cortical-lacunary in the deep territory of the MCA	No	Yes (limited left arm movement)	24	64	M

*Three patients were trained in the sub-acute phase and 2 patients in the chronic state*.

The study was carried out in the Neurology Clinic of the Rehabilitation Hospital of Iasi. Inclusion criteria were:

- Age between 18 to 70 years;
- Survivor of a stroke in the territory of the middle cerebral artery, with residual spastic hemiparesis;
- Chronic stroke (between 1 month to 5 years in our study);
- Upper limb deficits defined by the patient and investigator as disability in performing daily activities;
- Able to attend the research or rehabilitation center as required for the protocol.

We have excluded patients with significant speaking/understanding and/or cognitive disorders, pacemakers or other cardiac/cerebral/spinal cord implants which do not allow the use of Functional Electrical Stimulation (FES), as well as intense spasticity and/or wrist clonus, or upper limb deformities (viciously consolidated fractures).

### Data acquisition and experimental paradigm

Data were recorded from 64 electrode sites (Figure 1, right side). The sampling rate was 256 Hz. A bandpass filter with cutoff frequency at 0.5 and 30 Hz was applied. The method of common spatial patterns (CSP) was used for spatial filtering. The spatially filtered data were classified using the linear discriminant analysis (LDA).

**Figure 2:**
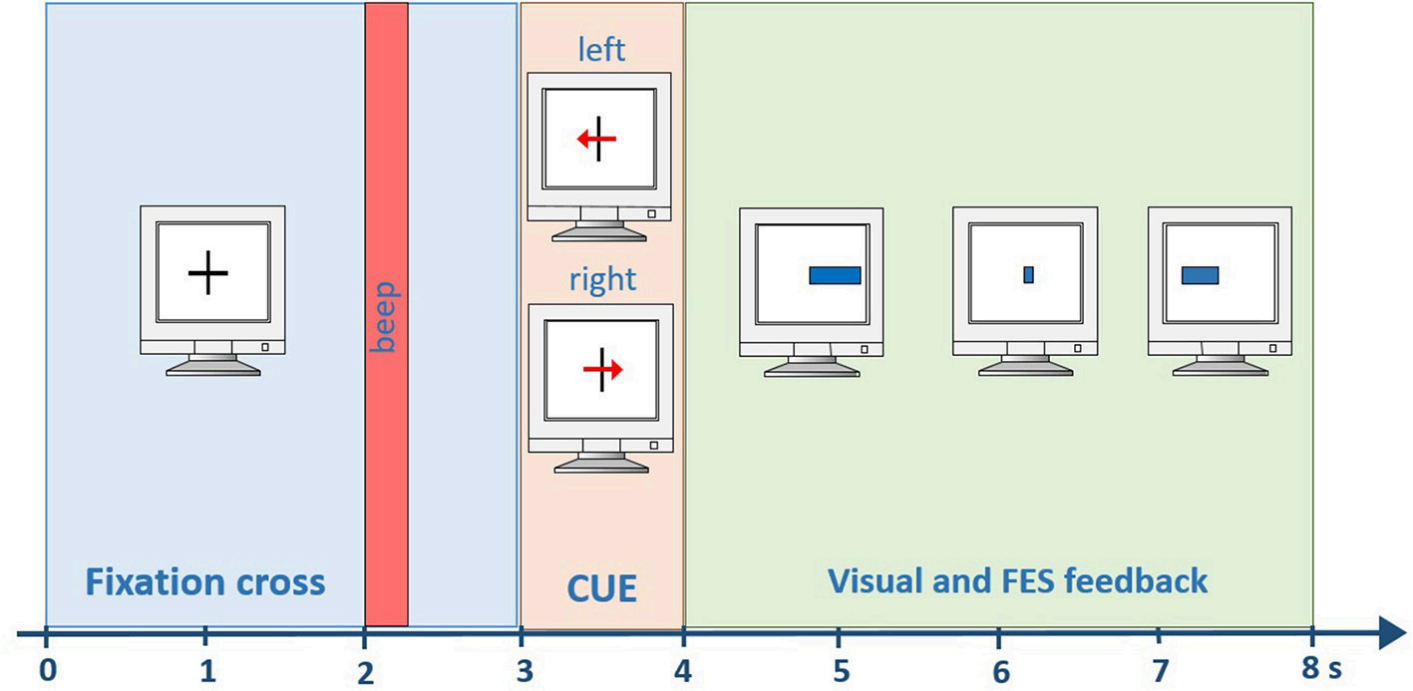
Timing within one trial. A beep indicates the start of the trial after 2 s. Then, a visual cue is presented for one second. From second four until the end of the trial (second 8) visual and FES feedback is provided to the user.

Each session began with mounting the electrode cap and FES pads. FES stimulation was provided through an 8-channel neurostimulator (MOTIONSTIM8, Krauth+Timmermann GmbH, Germany). The investigator adjusted FES parameters (pulse width and current) before each session to induce wrist dorsiflexion and hand opening without causing discomfort in both upper limbs. The most frequent setting was: healthy hand current ~15 mA, PW 300 us; affected hand: 20–22 mA, PW 300 μs. All patients participated in one practice session to become familiar with the system and feedback. During this practice session, patients were taught how to perform the MI task and performed two runs. The visual and FES feedback during the practice runs was artificially generated by the paradigm, and the recorded data was used as calibration data for the next session. Each run lasted 6 min and contained 40 MI trials (20 for each hand). Each trial lasted for 8 s with a 2 s break in-between. At the beginning of each trial, a cross was displayed in the center of the screen. After 3 s, an arrow pointed to the left or right side, providing the command to perform MI of either the left or right hand. One second later, the feedback bar appeared and was presented for four seconds. It showed the classified LDA distance, extending to the left for a negative LDA distance and to the right for a positive LDA distance (Figure 2).

**Figure 3:**
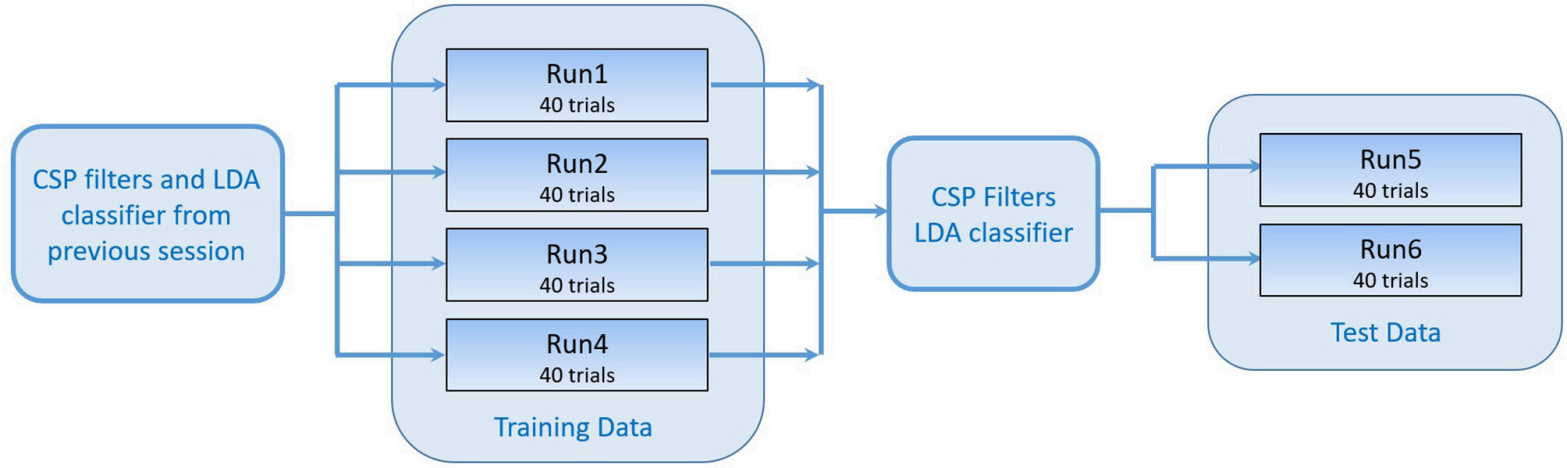
Workflow of one session. The training data consisting of 4 runs were recorded using the CSP filters and classifier generated in the previous session. The test data was used to generate a new set of CSP filters and LDA classifier that were tested online while recording runs 5 and 6.

A negative distance means that the left hand was classified, whereas a positive distance means the right hand was classified. In parallel, the FES system induced movements on the left or right hand, according to the classification result. A certain threshold had to be exceeded for a preceding time of 0.5 s to start stimulation. All subsequent (i.e., post-practice) sessions contained six runs in total, 4 runs as training data and 2 runs as test data (Figure 3). A short break was conducted between the runs.

With three of the patients (with less severe motor impairment), we were able to assess hand function using the “nine-hole PEG test” [9-HPT; (Croarkin et al., 2004)]. This test measures the time required to put 9 small metal pegs into a board with 9 holes, then remove the pegs. We also recorded the number of times a peg was dropped. The test is repeated for both hands.

Due to practical limitations in working with the patients (including both logistical (e.g., distance from study center and availability) and physical capacities), the total number of experimental sessions ranged from 10 to 24 across different participants. 9-HPT testing was performed after every third recording session with the three available cases. We also conducted testing using the Medical Research Council (MRC) guidelines for all five patients.

### Feature extraction and classification

The Common Spatial Patterns (CSP) approach is a frequently used algorithm in MI-based BCIs. It designs subject specific spatial filters resulting in maximized variance for one class of MI, and a minimized variance for the other class. Further explanations about the method of CSP could be found e.g., here (Mueller-Gerking et al., 1999; Ramoser et al., 2000; Blankertz et al., 2008). By spatially filtering the EEG data with the CSP filters, four features are created. These four features are further processed before classification is done. First, the variance (VARp) is calculated for a time window of 1.5 s. Second, they are normalized and log-transformed, as follows:

$$\begin{array}{l} x_{p}=log\left(\frac{VAR_{p}}{\sum_{p=1}^{4}VAR_{p}}\right) \end{array}$$

The vector of features is therefore: $\vec{x}={\left[x_{1}x_{2}x_{3}x_{4}\right]}^{T}$. A Fisher's linear discriminant analysis (LDA, Lemm et al., 2011) classification was done to categorize the data as either left hand MI or right hand MI. The discrimination function of the LDA is defined by a hyperplane, which is also called decision surface. The LDA distance $g\left(\vec{x}\right)$ is calculated as:

$$\begin{array}{l} g\left(\vec{x}\right)=w^{T}\vec{x}+w_{0} \end{array}$$

The plane is parametrized by its normal vector *w*^*T*^ and the bias *w*_0_ (Duda et al., 2000). *w*^*T*^ is also called the LDA weight vector. $\vec{x}$ represents the vector of features as defined above. The hyperplane divides the feature space into two half-spaces. The function $g\left(\vec{x}\right)$ gives a measure of the distance from a point $\vec{x}$ to the hyperplane. The distance can have a positive sign or a negative sign, the sign defines on which side of the hyperplane an observation lies. Therefore, $\vec{x}$ is classified as left hand MI if $g\left(\vec{x}\right)$ has a negative sign and as right hand MI otherwise. In the calibration phase, the training data (runs 1 to 4), was used to calculate 5 sets of spatial filters and classifiers from two-second time windows, shifted in time with a 0.5 s Hamming window based on data from the time interval from 4 to 8 s in each trial. The classifier with the highest ten-fold cross-validated accuracy (Naseer et al., 2014) was chosen to provide visual and FES feedback while recording runs five and six, which were used to calculate the online accuracy (*A*_*o*_) of the chosen classifier for the current session as follows:

$$\begin{array}{l} A_{o}=\frac{N_{CC}}{N}\times 100, \end{array}$$

where *N*_*CC*_ is the number of correctly classified trials and *N* is the total number of trials. While recording the first four runs, the feedback was provided using the spatial filters and classifier calculated in the previous session.

## Results

### Classification accuracy

**Figure 4:**
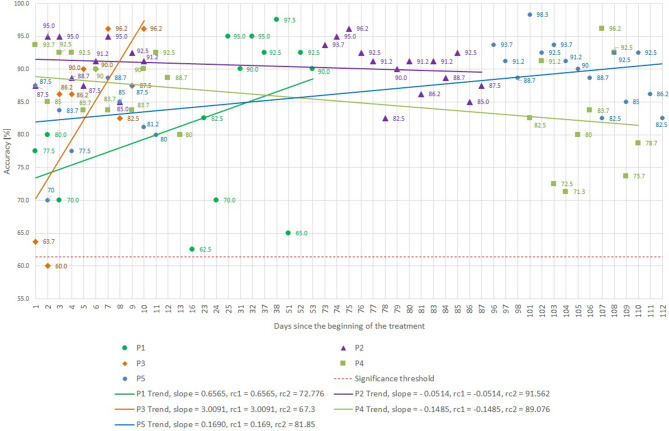
BCI accuracy attained for different subjects and sessions, as well as trendlines for each subject. The slope and regression coefficients (rcx) are presented for each trendline. Please note that the x-axis is not linear to highlight early and late training effects. The horizontal dashed red line reflects the significance threshold.

Figure 4 presents BCI control accuracy scores for all five patients, based on the online results (not *post-hoc* cross-validated data) achieved while recording runs five and six (the data from runs 1–4 was used to set up the CSP filter and to train the LDA), and the trend-lines of the accuracies in each patient. Patients P1, P2, P4, and P5 started with an accuracy above 70%. P3 began at only 61%, which is below the significance threshold of 61.4%, but improved rapidly. The threshold for significant accuracy depends on the total number of trials (Billinger et al., 2013; Yuan et al., 2013), and we developed the significance threshold of 61.4% for each run based on MATLAB's binofit function, which uses the Clopper-Pearson method for calculating the confidence intervals (Cruse et al., 2011).

Minimum, average, and maximum accuracies are shown in Table 2. All patients reached an average accuracy above or equal to 82.7% during the training, and patients P2 and P5 were above 90%. Every patient attained accuracy above or equal to 96.2% in at least one session. P1, P3, and P5 improved their accuracy with training, P2 consistently exhibited high accuracy, and P4's accuracy actually declined during training, though it did not vary much overall.

**Table 2:** The minimum, the average and the maximum accuracy and improvement for the patients.

**Patient**	**Minimum accuracy [%]**	**Average accuracy [%]**	**Maximum accuracy [%]**	**Accuracy improvement**	**Motor improvement**
P1	62.5	82.7	97.5	Yes	Yes
P2	82.5	90.3	96.2	No	Yes
P3	60	83.7	96.2	Yes	Yes
P4	71.3	85.6	96.2	No	Yes
P5	70	94.5	98.3	Yes	Yes
Total	69.3	87.4	96.9		

### Motor performance improvement

Table 3 presents the results of 9-hole PEG tests for P1, P2, and P5. The first line in the table, numbered as session zero, represents the baseline performance for that patient. P1's 9-hole PEG test time improved for both healthy and paretic hands. P2 and P5 improved with the paretic hand. The “total time improvement” row at the bottom of Table 3 shows that all three participants required less time to perform the 9-HPT with the paretic hand after training. P1 and P2 may have exhibited a “ceiling effect,” as their performance with the paretic hand became close (P1) or equal (P2) to the non-paretic hand.

**Table 3:** The results of the 9-hole PEG test for patients P1, P2, and P5.

**Sessions**	**Paretic hand Time [s]/dropped PEGs**			**Healthy hand Time [s]/dropped PEGs**		
	**P1**	**P2**	**P5**	**P1**	**P2**	**P5**
0 (baseline)	52/1	65/-		46/-	31/-
3	52/2	54/-		46/-	32/-
6	45/-	45/-		40/1	32/-
9	40/-	42/-	90/1	35/-	31/-	26/-
12	40/-	42/-	77/-	32/-	31/-	26/-
15	38/-	38/-	94/-	33/-	29/-	26/-
18		34/-	60/-		29/-	25/-
21		30/-	61/-		29/-	25/-
24		30/-	52/-		29/-	26/-
Total time improvement [s]	14	35	38	13	2	0

*The table contains the time to complete the test and dropped PEGs for the left and right hands for several sessions. The bottom row presents the total time improvement in seconds*.

Patient P5's condition didn't allow him to perform the 9-hole PEG test during the first training session. After 9 sessions of training, the motor functions of his left hand improved such that he managed to perform the task for the first time, which was designated as the baseline.

**Figure 5:**
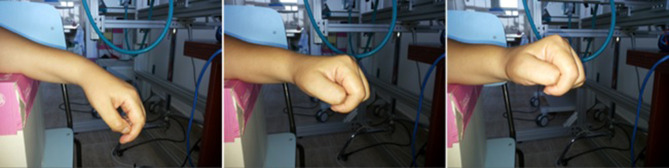
The voluntary wrist movements performed by patient P3 after 10 sessions of recoveriX training. Four years after the stroke the patient was not able to do any kind of voluntary movements with the hand and wrist, her arm being totally paralyzed from the elbow downwards. After the training, she was able to voluntary perform the wrist dorsiflexion on a range of about 45 degrees.

P3 could not perform the 9-hole PEG test because of the severity of the motor deficit. After the 10th session, she regained some voluntary control of wrist dorsiflexion, as shown in Figure 5, but no improvement in voluntary finger control.

P4 also could not perform the 9-hole PEG test throughout the study. He started to move his index finger and thumb after the 12th session (MRC scores of 1, Table 4), and the range of his fingers' voluntary movements increased after the 22nd session (MRC score of 2), but he did not achieve enough motor control to allow fine prehension.

**Table 4:** Medical Research Council scale (MRC) scores (range from 0 to 5) for different muscle groups of the affected upper limbs (before/after training).

**Patient**	**Elbow flexion**	**Elbow extension**	**Wrist flexion**	**Wrist extension**	**Finger flexion**	**Finger extension**
P1	4/4	4/4	4/4	4/4	4/4	4/4
P2	3/5	3/5	3/5	3/5	3/5	2/5
P3	2/2	2/2	0/1	0/2	0/0	0/0
P4	4/4	3/3	2/2	2/2	1/2	1/2
P5	4/5	3/4	3/5	3/4	3/5	3/4

## Discussion

The present results suggest that stroke patients can control a BCI with high accuracy even with the lesioned hemisphere, despite damage to motor areas and other potential problems (such as diminished attention, concentration, or motivation). Interestingly, the grand average accuracy of all 5 patients is 87.4% and the mean maximum accuracy is 96.9% across all subjects and sessions (10 to 24). This might be explained by the patients' high motivation to participate in the study. Patients reported that they were eager to come back for further recoveriX training because they could see a functional improvement.

In addition to motivating patients, the real-time feedback may have contributed in other ways. The feedback provided a means to coach the patients to perform the training optimally. P3 started with very poor classification accuracy and was encouraged to improve by the experimenter, which resulted in a clear improvement over training sessions. The feedback also provides an objective measure of task compliance, and can help maintain patients' attention. Feedback provided through visual stimuli and the FES system should better engage patients by engaging visual, tactile, and proprioceptive systems that feed back to the sensorimotor system. BCI-FES training has been shown to produce functional gains, but still large scale clinical trials are missing to show the efficacy (Young et al., 2014).

Another reason that tight coupling between each user's brain activity and feedback is important is that it can improve Hebbian learning, which is critical to motor recovery after stroke. This simultaneous activation is critical in Hebbian learning. Within this hypothesis, if the two areas are not simultaneously active—such as if the patient is not imagining the expected movement during some or all of the stimulation—then therapy benefits are reduced. BCI systems that activate feedback mechanisms in situations when MI is performed correctly should facilitate this simultaneous activation and thus improve therapy outcomes, as noted by several groups cited above. Indeed, further research exploring optimal adaptive parameters to best couple FES activation and other feedback with relevant CNS activity is warranted.

In 2015, our group performed a study on 20 healthy subjects using the same paradigm. The only difference in the experimental setup was the use of FES feedback only on stroke patients. All subjects operated the CSP-based BCI across one session. The grand average maximum accuracy was 80.7%, compared to 96.9% in this study. The grand average mean accuracy was 72.4%, compared to 87.4% in the current study. While that study used fewer training sessions than the current study, the mean accuracy in session 2 of the current study is 78%, which is still higher. The fact that all patients from the current study attained above 90% accuracy in at least one session could be a proof that many stroke patients can achieve very good classification accuracies.

In a study from 2000 (Guger et al., 2000), three subjects were trained over 6 or 7 sessions (2 without feedback and all others with feedback). This study used 27 electrodes with CSP and LDA like the current study. S1, S2, and S3 achieved 87.7, 74.0, and 77.5% mean accuracy across all feedback sessions. S1 and S2 continuously improved their performance to 98.2 and 93.2%. S3 showed some fluctuations and had the highest accuracy in session 2, but the last session was similar with 86% accuracy. In this case, 2 out of 3 patients achieved accuracy above 90%, but were trained less than in the current study.

Ang et al. (2011) compared the performance of 46 stroke patients to 16 healthy subjects (Ang et al., 2011), using filter bank CSPs for feature extraction. He found 6 out of the 46 patients to perform at chance level and the healthy controls to perform slightly better than patients. The average accuracy of the patients was 74 vs. 78% of controls. The BCI in this study classified active state vs. resting state, which could explain the worse accuracy compared to our results. It confirms though that most stroke patients have a good BCI control, a finding that is in coherence to our results.

Before this study, we hypothesized that it might be more difficult for the stroke patients to achieve high accuracies, but the patients performed very well. As shown in Table 3 and Figure 5, all patients did exhibit improvements in motor function, both in the sub-acute and chronic stage. Further research should parametrically compare improvements to conventional controls.

An imperfect classification accuracy is also not necessarily a problem for patient training. If a user reaches 90% accuracy, for example, then 90% of the paired-stimuli (brain and FES) are still provided correctly. This should be sufficient to convey a sense of control, and could also help users improve further.

It is also interesting to compare the performance of more recent vs. more chronic patients. Chronic patients P1 and P3 achieved a mean accuracy of 82.7 and 83.7%. Patients with more recent strokes (less than 3 months)—P2, P4, and P5—achieved 90.3, 85.6, and 94.5%. Therefore, it seems that it is easier for sub-acute patients to attain high accuracies, but of course more data is necessary to explore this issue further.

Patients P1, P2, and P5 had a cortical small lacunar stroke in the deep territory of the MCA, while P3 and P4 had a cortical superficial stroke. P5 outperformed all other patients in terms of accuracy and showed also the biggest improvement in the 9-hole PEG test (38 s), but more data would be necessary to make further conclusions.

We observed a non-continuous increase in control accuracy during the first sessions in only P3 and P5. Medication and fatigue could be factors that affect performance and performance variability. We did not parametrically assess the possible effects of these two factors, which would be of interest in future work. Nonetheless, accuracy can serve as an objective measure of whether patients are able and willing to perform MI tasks that are often required during therapy. Even without motor improvements, accuracy provides an important tool to inform the operator that the patient is participating. The precision of this detection remains an issue for further research; with current EEG methods, patients might be imagining slightly different movements than expected.

One of the possible confounders in our study is the lack of homogeneity of stroke patients. Further studies will have to explore patients with different sizes and locations and age of the ischemic lesion to develop more definitive results and further explore which patients would benefit from this technique. As a preliminary result, our study shows that the training had a favorable impact on patients with different types of strokes. Further work might elucidate how to best tailor therapy and expected outcomes for different patients.

We also found that older patients had difficulty with the bar feedback when classification was incorrect, because it was hard to associate the corresponding movement with the feedback. Some recent work has validated VR technology within the context of MI BCIs for stroke rehabilitation (Luu et al., 2015; Soekadar et al., 2015; Remsik et al., 2016; Sabathiel et al., 2016). Therefore, a newer version of the recoveriX system uses a virtual avatar, and the patient sees the left and right hands in a first-person perspective (Sabathiel et al., 2016). At the beginning of each trial, the left or right hand moves for 1 s, which triggers the patient to start the corresponding movement imagery. When the BCI system correctly classifies the activity, then the avatar hand movement is prolonged and the FES is triggered. When the classification is wrong, then the avatar and the FES are temporarily inactive. Future patients will be trained with this VR-based system. Future work needs to further explore the relative contributions of different types of feedback, including FES activation and visual feedback such as bar feedback and avatars, which cannot be definitively answered from the present study.

Most importantly, these and other questions need to be further explored with larger groups of patients. In 2015, our group performed a study on 20 healthy subjects using the same paradigm on the same hardware system (Ortner et al., 2015). The only difference in the experimental setup was the use of FES feedback only on stroke patients. While new research shows that active FES mainly contribute to cortical reorganization, expressed in motor improvements as tested after the BCI sessions (Quandt and Hummel, 2014), the focus of the main experimental session was to determine the level of classification accuracy for stroke patients and to compare it with the one from previous study on healthy subjects. Indeed, further work may explore at which degree the effect of the neurofeedback (either visual or FES) influences or not the classification accuracy during a session or over the entire experiment on a subject. The current results are presented as a limited case study with only five patients. The small sample size and the absence of a control group represent the main drawbacks of this work. At the same time, these facts limit the opportunities for statistical testing and exploring differences within patient groups (such as sub-acute vs. chronic), and the nature of the indirect comparison with another study is inconclusive.

## Author contributions

DI contributed to the conception of the work, data acquisition, analysis and interpretation of data, and drafted the manuscript. RO contributed to the conception of the work, interpretation of the data, and revised the work critically. MP contributed to the conception of the work, interpretation of the data and revised the work critically. BI contributed to the conception of the work, *in-vivo* experiments supervision, interpretation of the data, and revised the work critically. CG contributed to the conception of the work, analysis and interpretation of the data, and revised the work critically. Furthermore, all authors gave final approval for the version to be published and agreed to be accountable for all aspects of the work.

### Conflict of interest statement

The authors declare that the research was conducted in the absence of any commercial or financial relationships that could be construed as a potential conflict of interest.
